# Substance Use Behaviors as Markers of Emotional Dysregulation and Childhood Adversity Among Adult Emergency Department Patients

**DOI:** 10.7759/cureus.101182

**Published:** 2026-01-09

**Authors:** Amar Kassim, Abdulla Sabry, Christina Schweitzer, Rosemarie Burynski, Alexandra DiGiovanni, Doi Bu, Meg Russell, Stephen Sandelich

**Affiliations:** 1 Urology, Penn State College of Medicine, Hershey, USA; 2 Internal Medicine, Penn State College of Medicine, Hershey, USA; 3 Anesthesiology, Penn State College of Medicine, Hershey, USA; 4 Neurosurgery, Penn State College of Medicine, Hershey, USA; 5 Pediatric Emergency Medicine, Penn State Health Milton S. Hershey Medical Center, Hershey, USA

**Keywords:** adverse childhood experiences, behavioral health, cognitive reappraisal, emergency department, emotional dysregulation, substance use, trauma-informed care

## Abstract

Introduction

Substance use is frequently encountered in emergency department (ED) visits and may reflect underlying emotional or developmental challenges. Adverse childhood experiences (ACEs) are linked to both emotional dysregulation and later substance use, but this relationship is rarely examined in acute care settings. We investigated whether recent substance use, as measured by the Tobacco, Alcohol, Prescription medication, and other Substance use (TAPS) screening tool, is associated with ACEs, emotional dysregulation, and use of cognitive reappraisal (an adaptive emotion regulation strategy) among adult ED patients.

Methodology

In this cross-sectional study, 58 adult ED patients completed the ACE Inventory, the Difficulties in Emotion Regulation Scale (DERS), the Emotion Regulation Questionnaire (ERQ)-Cognitive reappraisal subscale, and the TAPS screening. Descriptive statistics characterized the sample. Pearson correlations and linear regression analyses (adjusting for age and gender) examined associations between TAPS scores and ACE, DERS, and ERQ scores.

Results

TAPS scores were positively correlated with ACEs (*r* = 0.23, *P* < 0.05) and DERS scores (*r* = 0.30, *P* < 0.01), and negatively correlated with ERQ-Cognitive reappraisal scores (*r* = -0.22, *P* < 0.05). In regression models, higher TAPS scores significantly predicted greater ACE burden, higher DERS scores, and lower ERQ-Cognitive reappraisal scores (all *P* < 0.05, with covariate adjustment). Participants reporting alcohol or marijuana use had significantly higher mean ACE scores than non-users.

Conclusion

Recent substance use identified during ED visits was associated with greater childhood trauma exposure and poorer emotion regulation. Substance use in the ED may serve as a marker of underlying emotional dysregulation and adversity. These findings support the incorporation of trauma-informed screening and referral practices into ED care as part of integrated care models to better address these underrecognized psychosocial factors.

## Introduction

Substance use is frequently encountered in the emergency department (ED), with nearly one in eight ED visits in the United States involving issues related to alcohol, illicit substances, or the non-medical use of prescription substances [1]. The ED often acts as a critical access point for patients who may face barriers to routine medical or mental health care, making it an essential setting for identifying behavioral health concerns that might otherwise go unaddressed. Notably, national data show that ED visits related to substance use are most commonly driven by stimulant-, sedative- (including opioid and benzodiazepine), and hallucinogen-related presentations, rather than cannabis alone [1]. Our study was conducted in a suburban hospital in Pennsylvania during a period when medical cannabis was legal but adult recreational use was restricted, which may help explain differences in the types of substances reported by participants and underscores that patterns of substance use vary considerably by region and legal environment.

Substance use is increasingly understood not only as an isolated behavior but also as a potential indicator of deeper emotional and developmental challenges. A large body of research has linked Adverse Childhood Experiences (ACEs) to both emotional dysregulation and later-life substance use [2-4]. Within a trauma-informed and emotion regulation framework, early adversity is theorized to disrupt neurobiological stress and regulatory systems, thereby increasing vulnerability to maladaptive coping behaviors in adulthood. Emotional dysregulation, defined as difficulty identifying, tolerating, and modulating emotional responses, may represent a key pathway by which early trauma contributes to maladaptive coping behaviors such as substance misuse [5]. For instance, severe or chronic trauma exposure in childhood has been associated with complex developmental psychopathology and impaired regulatory capacity, sometimes conceptualized within developmental trauma models [6]. Despite these connections, ED assessments rarely integrate questions about trauma history or emotional regulation into standard substance use screening [7].

Validated measures such as the Difficulties in Emotion Regulation Scale (DERS) and the Emotion Regulation Questionnaire (ERQ) have demonstrated robust associations between ACE exposure, impaired emotion regulation, and substance-related outcomes across clinical and community samples [8-11]. Individuals with high ACE burdens may rely on substances to achieve rapid emotional relief when adaptive strategies such as cognitive reappraisal are less accessible. Despite this well-established theoretical and empirical foundation, ED assessments rarely integrate trauma history or emotion regulation capacity into routine substance use screening, even though trauma exposure is common among ED patients and associated with recurrent utilization and poor engagement with outpatient care [7].

Tools like the Tobacco, Alcohol, Prescription medication, and other Substance use (TAPS) tool are commonly used to identify patterns of recent substance use [9]; however, they are not routinely interpreted within the context of broader psychosocial vulnerability. Few studies have examined how current substance use behaviors, emotion regulation patterns, and ACE exposure intersect in the ED setting [7].

Guided by a trauma-informed and emotion regulation framework, this study seeks to investigate whether substance use, as captured by TAPS scores, reflects underlying emotional and developmental vulnerabilities among adult patients in the ED. Specifically, we assess whether higher TAPS scores are associated with (1) a greater burden of ACEs, (2) increased emotional dysregulation as measured by the Difficulties in Emotion Regulation Scale (DERS) [10], and (3) lower use of adaptive cognitive emotion regulation strategies based on the Emotion Regulation Questionnaire (ERQ-Cognitive) [11]. These relationships are evaluated while adjusting for demographic factors such as age and gender. We hypothesized that higher TAPS scores would correlate positively with ACE and DERS scores and negatively with ERQ-Cognitive reappraisal scores.

## Materials and methods

This cross-sectional observational study was conducted to determine the relationship between substance use and ACEs. The study was approved by the Institutional Review Board, and all participants provided informed consent before participation. The study is reported according to STROBE guidelines [8].

Study design and setting

The study was conducted at a single suburban ED between August 2024 and March 2025. Participants were recruited during their ED evaluation through a convenience sample of both weekends and weekdays.

Participants

Adults aged 18 years and older who presented to the ED for an acute medical evaluation were screened for eligibility. Inclusion criteria included primary English language proficiency, not currently incarcerated, medically stable (as defined by the treatment team), and able to provide informed consent. Patients were excluded if they were under 18 years of age, pregnant, or demonstrated cognitive impairment. Eligible patients were identified through review of ED records.

Importantly, participants were recruited irrespective of substance use status or psychiatric presentation. Most were medically stable individuals presenting for evaluation of diverse acute medical issues (e.g., injuries, infections) rather than substance-related or mental health concerns. Consequently, the study population included adults who might report past or current substance use without experiencing acute distress or interference in their lives. We did not administer additional measures of depression, anxiety, or suicidality; therefore, the severity of psychiatric symptoms and the functional impact of substance use cannot be ascertained from these data.

Measures

Recent substance use behavior was assessed using the TAPS tool, a validated screening instrument developed by the National Institute on Drug Abuse [9]. The TAPS tool evaluates past three-month use of multiple substance categories, including tobacco, alcohol, marijuana, prescription sedatives (e.g., benzodiazepines), prescription stimulants (e.g., amphetamines), and other illicit substances. Responses are scored on an ordinal scale reflecting frequency of use, with higher scores indicating more frequent or hazardous use. It is important to note that the TAPS assesses only current (past three‑month) use; individuals with a history of dangerous substance use who were currently abstinent would not be identified by this instrument.

Cumulative exposure to childhood adversity was measured using the ACE Inventory, a 10-item screening tool developed by the CDC-Kaiser Permanente ACE Study [2]. The ACE Inventory captures exposure to physical, emotional, and sexual abuse, neglect, and household dysfunction occurring before the age of 18. Each endorsed experience contributes one point to the total score, which ranges from 0 to 10. In accordance with established thresholds, scores were also stratified into low (<4) and high (≥4) ACE burden for subgroup analyses.

Emotional dysregulation was assessed using the Difficulties in Emotion Regulation Scale (DERS), a 36-item self-report measure evaluating six domains of emotion regulation difficulty: nonacceptance of emotional responses, difficulty engaging in goal-directed behavior, impulse control difficulties, lack of emotional awareness, limited access to emotion regulation strategies, and lack of emotional clarity [10]. Each item is rated on a 5-point Likert scale from 1 (*almost never*) to 5 (*almost always*), with higher total scores indicating greater dysregulation.

Cognitive emotion regulation strategies were measured using the Emotion Regulation Questionnaire (ERQ), a widely used 10-item self-report tool developed by Gross and John [11]. This study focused on the ERQ’s cognitive reappraisal subscale, which includes six items assessing the tendency to reinterpret situations in ways that alter their emotional impact. Items are rated on a 7-point Likert scale ranging from 1 (*strongly disagree*) to 7 (*strongly agree*), with higher scores reflecting greater use of adaptive cognitive reappraisal strategies.

The study instruments, including the ACE questionnaire, the DERS, and the ERQ, were administered under a protocol approved by the Penn State Institutional Review Board, which authorized their use for academic research purposes in a non-commercial context. Additionally, since DERS and ERQ are not open-access, primary authors were contacted to obtain permission to use the scales.

Data collection

After identifying eligible patients, research staff obtained written informed consent (including HIPAA authorization) via REDCap on an iPad. Participants then completed the ACE, DERS, ERQ, and TAPS measures independently using the REDCap program. No identifying information was linked to survey responses, which were stored under a de-identified study ID.

Demographic and clinical data were extracted from the electronic medical record. Descriptive statistics were used to characterize the sample, as displayed in Table 1. Group comparisons were conducted to examine differences in ACE scores by substance use status. Pearson correlations were used to assess associations between substance use (TAPS) and ACE, DERS, and ERQ scores. Multiple linear regression models were performed to determine whether TAPS scores predicted ACE burden, emotional dysregulation, and cognitive reappraisal, adjusting for age and gender. Missing data were minimal and limited to demographic variables (gender: n = 5, 8.6%; race: n = 1, 1.7%; marital status: n = 1, 1.7%). No missing data were present for primary study variables (TAPS, ACE, DERS, or ERQ scores). Analyses were therefore conducted using complete-case (listwise) deletion for models including demographic covariates. Assumptions underlying linear regression, including linearity, normality of residuals, homoscedasticity, and absence of multicollinearity, were evaluated and deemed acceptable based on visual inspection of residual plots and variance inflation factors. All analyses were conducted by institutional statisticians using IBM SPSS Statistics, version 29.0 (IBM Corp., Armonk, NY). To minimize interviewer bias, all surveys were self-administered on tablet devices in private ED rooms.

The target sample size was determined based on prior published effect estimates demonstrating moderate associations between substance use and emotion regulation (*r* ≈ 0.4), indicating that a minimum of 46 participants would provide 80% power to detect statistically significant correlations at α = 0.05. This sample size is also consistent with commonly cited recommendations for linear regression analyses, which suggest a minimum of 10-15 observations per predictor variable. Given that regression models included one primary predictor (TAPS score) and two covariates (age and gender), the final sample size of 58 participants was deemed sufficient to support adequately powered regression analyses while minimizing model overfitting.

## Results

As depicted in Table 1, a total of 58 participants (100%) were included in the final cohort. The mean age was 46.2 years (SD = 17.4). The sample included 39 females (67.9%), 19 males (32.1%), with gender missing for 5 participants (8.6%). The majority of participants were White (*n* = 35, 70.0%), followed by Black (*n* = 6, 12.0%) and Other/Multiracial (*n* = 9, 18.0%); race data were missing for one participant (1.7%). Regarding marital status, 30 participants were single (51%), 22 were married (37%), and 7 were divorced, separated, or widowed (12%), with one missing response (1.7%). All correlation and regression analyses retained the full sample for primary outcome measures, as no missing data were present for TAPS, ACE, DERS, or ERQ scores.

**Table 1:** Baseline demographic and clinical characteristics of the study sample. Values are reported as *n* (%) for categorical variables and mean ± standard deviation (SD) for continuous variables. ACE, Adverse Childhood Experiences; DERS, Difficulties in Emotion Regulation Scale; ERQ, Emotion Regulation Questionnaire

Variable	Value
Sample size	58 (100%)
Age (years), mean ± SD	46.2 ± 17.4
Female	39 (67.9%)
Male	19 (32.1%)
Gender missing	5 (8.6%)
White	35 (70.0%)
Black	6 (12.0%)
Other/Multiracial	9 (18.0%)
Race missing	1 (1.7%)
Single	30 (51.0%)
Married	22 (37.0%)
Divorced/Separated/Widowed	7 (12.0%)
Marital status missing	1 (1.7%)
ACE score, mean ± SD	2.74 ± 2.46
DERS score, mean ± SD	34.7 ± 13.1
ERQ-Cognitive reappraisal score, mean ± SD	28.8 ± 11.1

Descriptive analyses indicated a mean ACE score of 2.74 (SD = 2.46, range 0-8), a mean DERS score of 34.7 (SD = 13.1, range 0-61), and a mean ERQ-Cognitive reappraisal score of 28.8 (SD = 11.1, range 0-42). Gender, race, and marital status were not significantly associated with TAPS, ACE, DERS, or ERQ-Cognitive reappraisal scores. Age showed a weak, non-significant positive correlation with TAPS scores. Notably, none of the participants reported use of opioids, cocaine, or methamphetamines during the study period. This absence likely reflects the suburban catchment area and the state’s legal landscape regarding controlled substances and cannabis. Consequently, our sample may not be representative of EDs that see higher volumes of opioid or stimulant presentations, and caution is warranted when generalizing these findings to other settings.

**Figure 1:**
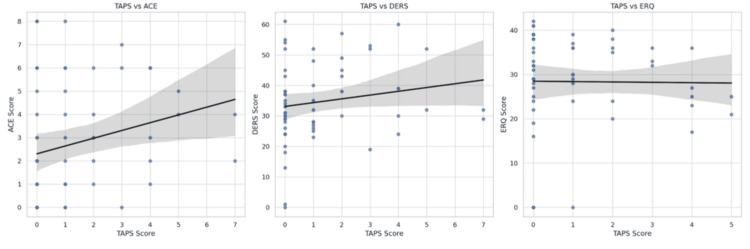
Correlation plots (ACE, DERS, and ERQ against TAPS). ACE, Adverse Childhood Experiences; DERS, Difficulties in Emotion Regulation Scale; ERQ, Emotion Regulation Questionnaire; TAPS, Tobacco, Alcohol, Prescription medication, and other Substance use

Correlation analyses revealed that TAPS scores were positively correlated with ACE scores (*r* = 0.23, *P* < 0.05) and DERS scores (*r* = 0.30, *P* < 0.01), and negatively correlated with ERQ-Cognitive reappraisal scores (*r *= -0.22, *P *< 0.05), as demonstrated in Figure 1. These results suggest that higher levels of recent substance use were associated with greater childhood adversity, greater emotional dysregulation, and lower use of cognitive reappraisal strategies. These correlations are summarized in Table 2.

**Table 2:** Correlation matrix (TAPS, ACE, DERS, and ERQ-Cognitive). ACE, Adverse Childhood Experiences; DERS, Difficulties in Emotion Regulation Scale; ERQ, Emotion Regulation Questionnaire; TAPS, Tobacco, Alcohol, Prescription medication, and other Substance use

Variable pair	r	*P*-value
TAPS and ACE	0.23	<0.05
TAPS and DERS	0.30	<0.01
TAPS and ERQ-Cognitive reappraisal	−0.22	<0.05

Linear regression models confirmed these associations (Table 3). TAPS scores significantly predicted ACE scores (unstandardized β > 0, *P* < 0.05), DERS total scores (β > 0, *P* < 0.01), and ERQ-Cognitive reappraisal scores (β < 0, *P* = 0.03). All relationships remained statistically significant after adjusting for age and gender, indicating strong links between substance use and both childhood adversity and emotion regulation functioning.

**Table 3:** Regression models predicting ACE, DERS, and ERQ from TAPS. ACE, Adverse Childhood Experiences; DERS, Difficulties in Emotion Regulation Scale; ERQ, Emotion Regulation Questionnaire; TAPS, Tobacco, Alcohol, Prescription medication, and other Substance use

Outcome variable	β (Unstandardized)	*P*-value
ACE score	+	<0.05
DERS total score	+	<0.01
ERQ-Cognitive reappraisal score	-	0.03

**Figure 2:**
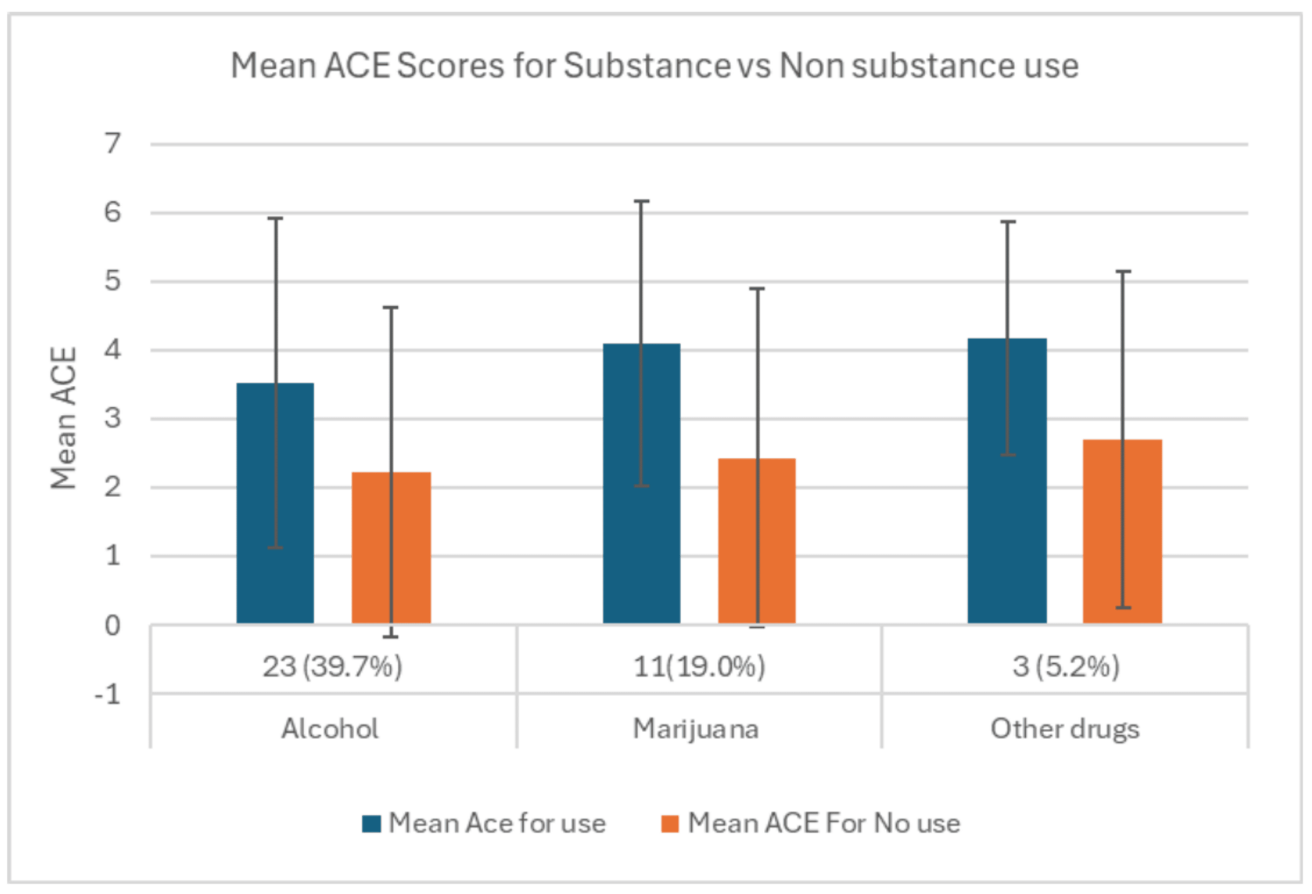
Comparison of reported substance use across the mean ACEs. ACE, Adverse Childhood Experiences

To explore patterns of substance use by type, participants were divided into use versus non-use groups for alcohol, marijuana, and a combined *other drug* category (benzodiazepines and stimulants combined for statistical power due to low *n*), as shown in Table 4. Alcohol use (*n* = 23, 39.7%) was associated with higher ACE scores (mean = 3.52, SD = 2.39) compared to non-users (mean = 2.23, SD = 2.40; *P* = 0.05). Marijuana users (*n* = 11, 19.0%) also reported significantly higher ACE scores (mean = 4.09, SD = 2.07) than non-users (mean = 2.43, SD = 2.46; *P *= 0.033). Participants in the *other drug* category (*n* = 3, 5.2%) had higher ACE scores (mean = 4.17, SD = 1.70) than those not reporting other drug use (mean = 2.69, SD = 2.45), although this difference did not reach statistical significance (*P* = 0.157). Figure 2 illustrates the mean ACE scores among users and non-users for each substance category.

**Table 4:** Substance use stratified by ACE scores. Other drugs = Stimulants + Benzodiazepines (combined for statistical power due to low *n*) ACE, Adverse Childhood Experiences

Substance	Reporting use, *n* (%)	Mean ACE for use	Mean ACE for no use	*P*-value
Alcohol	23 (39.7%)	3.52 (SD = 2.39)	2.23 (SD = 2.4)	0.05
Marijuana	11 (19%)	4.09 (SD = 2.07)	2.43 (SD = 2.46)	0.033
Other drugs	3 (5.2%)	4.17 (SD = 1.70)	2.69 (SD = 2.45)	0.157

## Discussion

This study adds to the growing body of evidence suggesting that substance use behavior is a potential behavioral marker of emotional dysregulation, particularly among individuals with a history of ACEs. Our findings are consistent with prior research showing strong associations between ACEs, impaired emotional regulation, and substance use [2-6]. The ACE framework provides a critical lens for understanding how early adversity shapes long-term behavioral and emotional outcomes. Previous literature has demonstrated that trauma in childhood can dysregulate the hypothalamic-pituitary-adrenal (HPA) axis, impacting emotional control and increasing vulnerability to maladaptive coping strategies such as impulsivity and substance dependence [3-5,12-15].

In exploratory substance-specific analyses, participants reporting alcohol or marijuana use demonstrated significantly higher ACE scores compared to non-users. The combined *other drug* category (benzodiazepines and stimulants) was not statistically significant, which may reflect the fact that two distinct substance types were pooled due to low frequency, potentially obscuring substance-specific associations.

The association between higher ACE scores and emotional dysregulation is corroborated by studies using validated scales like the DERS and the ERQ [11,12,14]. These tools reliably distinguish levels of emotional regulation across populations. Notably, emotional dysregulation may serve as a bridge linking ACEs to risky coping behaviors. Our findings that substance-using individuals scored higher on both the DERS and ERQ suppression subscales suggest that difficulties with identifying and modulating emotional states may contribute to substance use behaviors [5,6,13].

This is consistent with previous work identifying impulsivity as a maladaptive emotion regulation strategy among trauma-exposed populations [5,13,15]. Individuals with post-traumatic stress disorder (PTSD) or high ACE burdens may use substances to achieve rapid emotional relief, even at the cost of long-term harm [13,15,16]. Moreover, our study expands on prior findings by highlighting the unique patterns observed in an ED setting - a context often underrepresented in emotion regulation research [7,10,16-18].

Our findings also align with a trauma-informed perspective, which frames adult behavioral health symptoms as adaptive responses to earlier trauma. The ED, where patients frequently present with behavioral symptoms and comorbid substance use, represents a particularly relevant setting for applying this lens [17-19]. Studies show that trauma-related emotional dysregulation mediates health risks ranging from suicidality to substance misuse, further supporting the need for emotion-focused and resilience-building interventions [14,15,20].

Importantly, trauma-informed care offers an actionable framework. The Substance Abuse and Mental Health Services Administration (SAMHSA) advocates for universal trauma screening and systems-level change to address trauma at every point of care [21]. Our findings support this model and echo studies that show the benefits of integrating trauma-informed principles into acute care, including reductions in ED utilization and improvements in patient trust and engagement [22].

Future research should prioritize longitudinal evaluation of trauma-informed interventions tailored to emotionally dysregulated, substance-using individuals in emergency settings. Integrating ACE screening tools and brief emotion regulation assessments at triage may allow earlier identification of high-risk patients and facilitate timely referrals to behavioral health resources. Addressing substance use through a trauma-emotion framework could have meaningful implications for healthcare utilization, costs, and long-term outcomes among vulnerable populations.

## Conclusions

This study contributes to a growing body of research indicating that substance use behavior in ED patients can reflect deeper emotional and developmental challenges. Our results demonstrate that higher TAPS scores are significantly associated with greater ACE exposure and elevated emotional dysregulation. Substance use was inversely related to the use of cognitive reappraisal strategies, a key component of adaptive emotion regulation. These findings reinforce the idea that substance use in acute care settings is often a visible marker of underlying psychosocial distress, including trauma history and difficulty managing emotions.

These insights should inform future research and interventions aimed at addressing childhood adversity, ultimately improving care strategies and outcomes for future generations. Given the strong associations observed between substance use, ACEs, and emotional dysregulation, there is an opportunity to leverage ED substance use screenings to prompt trauma-informed interventions and appropriate behavioral health referrals. The ED setting represents a unique point of contact for individuals who may be disconnected from routine care. Incorporating trauma-informed screening and response protocols could help identify high-risk patients and connect them to appropriate support services. Future models of integrated care should consider embedding behavioral health resources into ED workflows to improve early identification and intervention for patients with significant emotional vulnerabilities.
